# Mechanisms of macular edema

**DOI:** 10.3389/fmed.2023.1128811

**Published:** 2023-03-07

**Authors:** Cameron D. Haydinger, Lisia Barros Ferreira, Keryn A. Williams, Justine R. Smith

**Affiliations:** Flinders University College of Medicine and Public Health, Adelaide, SA, Australia

**Keywords:** human, retina, macula, macular edema, diabetic retinopathy, retinal vein occlusion, uveitis

## Abstract

Macular edema is the pathological accumulation of fluid in the central retina. It is a complication of many retinal diseases, including diabetic retinopathy, retinal vascular occlusions and uveitis, among others. Macular edema causes decreased visual acuity and, when chronic or refractory, can cause severe and permanent visual impairment and blindness. In most instances, it develops due to dysregulation of the blood-retinal barrier which permits infiltration of the retinal tissue by proteins and other solutes that are normally retained in the blood. The increase in osmotic pressure in the tissue drives fluid accumulation. Current treatments include vascular endothelial growth factor blockers, corticosteroids, and non-steroidal anti-inflammatory drugs. These treatments target vasoactive and inflammatory mediators that cause disruption to the blood-retinal barrier. In this review, a clinical overview of macular edema is provided, mechanisms of disease are discussed, highlighting processes targeted by current treatments, and areas of opportunity for future research are identified.

## 1. Introduction

Macular edema is the pathological accumulation of fluid in the macula, the central region of the retina essential for high-acuity vision. It is a complication common to many ocular diseases. Fluid can accumulate diffusely in the central retina or within cysts usually localized in the inner nuclear layer or Henle’s fiber layer, or in the subretinal space, disrupting and distorting the retinal architecture and decreasing visual acuity (1, 2). In combination with the underlying pathology, the edema can progress to cause irreversible tissue damage with death of retinal cells, and permanent visual impairment. The common feature of most diseases that cause macular edema is dysfunction of the blood-retinal barrier (BRB), a set of structures that, in health, strictly regulates the passage of proteins, salts, metabolites and other solutes between the blood and the retinal tissue. Vasoactive growth factors and inflammatory cytokines, including vascular endothelial growth factor-A (VEGF) and tumor necrosis factor (TNF)-α, are upregulated in the ocular fluids in macular edema and have been implicated in disruption of the BRB. Dysregulated entry and accumulation of solutes in the retina disturb the balance of osmotic and hydrostatic forces, and lead to fluid entry when mechanisms maintaining fluid homeostasis are overcome. Current treatments target proteins and processes that are involved in angiogenesis, inflammation and blood-retinal barrier (BRB) dysfunction. This review provides an overview of clinical aspects of macular edema and discusses cellular and molecular mechanisms that are targeted by current treatments.

## 2. Clinical overview of macular edema

### 2.1. Associations and clinical features

Macular edema is a common feature of diverse eye diseases, such as diabetic retinopathy, retinal vascular occlusions, uveitis, post-cataract surgery inflammation (pseudophakic macular edema or Irvine-Gass syndrome), retinal dystrophies, drug reactions, intraocular tumors, serous central chorioretinopathy, radiation retinopathy and other retinal vascular abnormalities including retinal arterial macroaneurysms and retinal telangiectasia (3). Subretinal neovascular membranes observed in conditions such as age-related macular degeneration (AMD) are also associated with intraretinal and subretinal fluid accumulation (4).

**Figure 1:**
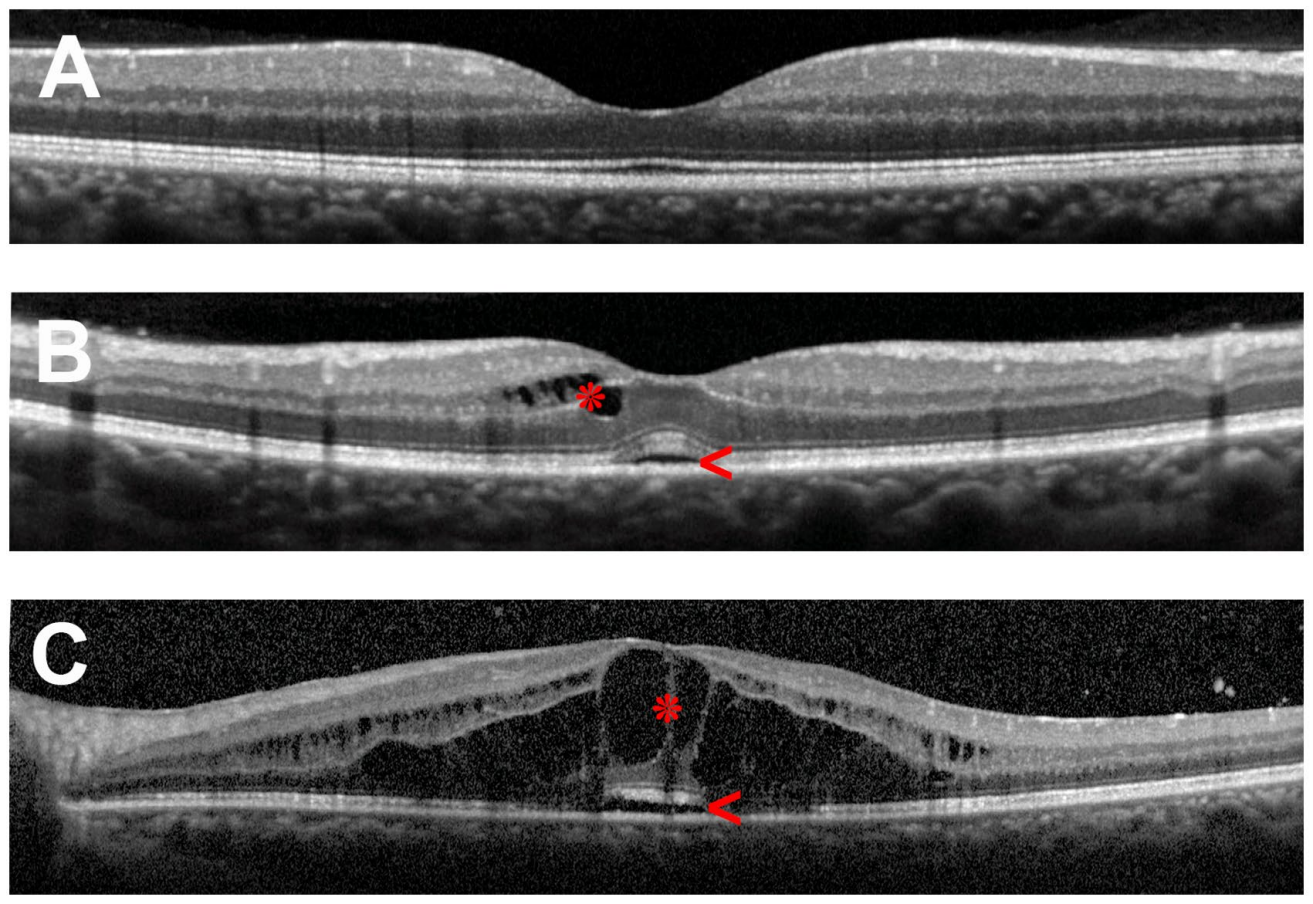
Optical coherence tomography (OCT) scans of the human macula in cross-section. **(A)** Healthy macula. **(B)** Macula of a patient with mild uveitic macular edema showing small numbers of cystic spaces in the inner retina (asterisk) and limited subretinal fluid (arrowhead). **(C)** Macula of a patient with severe uveitic macular edema demonstrating extensive retinal cystic changes (asterisk) and subretinal fluid (arrowhead).

Symptoms of macular edema include metamorphopsia, micropsia, blurred vision, a central scotoma, and reduction of contrast or color sensitivity. The clinical diagnosis of macular edema can be challenging in mild cases or when visualization of the fundus is impaired by poor pupillary dilation, cataract and other ocular media opacities. Therefore, fluorescein angiography and optical coherence tomography (OCT) are commonly employed to make the diagnosis. The latter is preferred due to its non-invasive nature, accurate measurements of retinal thickness and capacity for identifying other structural abnormalities, such as epiretinal membrane and vitreomacular traction, and it is the standard method for evaluating macular edema in clinical trials. Figure 1 illustrates the retinal changes that are observed by OCT in macular edema.

The thickness of the retina is generally measured between the inner limiting membrane and Bruch’s membrane. By current spectral-domain OCT technology, the average and central (foveal) macular thicknesses in normal adult eyes are 334 and 226 μm, respectively, being thicker in males than in females (5). Three patterns of macular edema have been described using OCT: cystoid macular edema, diffuse macular edema, and subretinal detachment (6, 7). Diffuse retinal thickening is the main pattern in diabetic macular edema (4, 6), while in retinal vascular occlusions, accumulation of subretinal fluid is common (8). In uveitis, cystoid and diffuse macular edema are the typical forms of macular edema, whereas subretinal detachment is seen in less than one-third of cases, usually in combination with cystoid or diffuse edema (7, 9, 10).

### 2.2. Burden of disease

The distortion of the retinal architecture caused by fluid accumulation in the macula results in vision loss, and potentially irreversible visual impairment due to scarring when the condition becomes chronic. Macular edema is the main cause of vision loss in patients with diabetes mellitus (11), retinal vein occlusions (12, 13), and uveitis (14, 15). Populational studies have shown a mean prevalence of diabetic macular edema of around 6% in type 1 diabetes, and slightly higher in type 2 diabetes (11). Macular edema is extremely common in central retinal vein occlusions, while in branch retinal vein occlusion, macular edema occurs in 30% of cases (16, 17). Around one-third of patients with uveitis develop macular edema, being more frequent in intermediate uveitis and panuveitis, and less common in anterior uveitis (14, 15). The burden of macular edema not only relates to the decrease in quality of life experienced by patients, but also to the chronicity of the condition and the economic costs associated with treatment and frequent visits to medical centers (18–20). In uveitic macular edema in particular, the most affected population group is of working age, adding to the socioeconomic impact of the condition (21–23).

### 2.3. Current treatment

Treatment of macular edema depends on the underlying cause. The front-line therapy of macular edema associated with diabetic retinopathy or retinal vascular occlusions is anti-VEGF therapy, delivered into the eye by intravitreal injection and sometimes augmented with intravitreal corticosteroid and/or retinal laser photocoagulation (24–26). Ranibizumab, a recombinant humanized antibody fragment against VEGF, and aflibercept, a recombinant protein comprising the ligand-binding domains of VEGF receptors 1 and 2 fused to the Fc portion of immunoglobulin G1, are FDA-approved anti-VEGF drugs, while bevacizumab, a humanized monoclonal antibody against VEGF, is widely used off-label. As well as VEGF, aflibercept binds to placental growth factor (PlGF). The treatment of macular edema secondary to neovascular AMD also relies heavily on the use of anti-VEGF therapy (27). Newer anti-VEGF drugs, brolucizumab and faricimab, have recently been approved for the treatment of neovascular age-related macular degeneration, and faricimab is also approved for use in diabetic macular edema (28, 29). Potential complications of anti-VEGF therapy include infectious endophthalmitis, sterile intraocular inflammation, and retinal vasculitis particularly with brolucizumab (30, 31). However, a population-based cohort study did not find any increase in the risk of myocardial infarction or cerebrovascular accidents in patients treated for neovascular AMD (32). Among the intravitreal corticosteroid therapeutics, intravitreal injections of triamcinolone acetonide and the dexamethasone ‘Ozurdex’ implant may be given as adjunctive treatments.

In contrast, corticosteroids are the mainstay for managing macular edema in non-infectious uveitis. Corticosteroid eye drops may be used if macular edema is mild and associated with anterior uveitis. Among the topical corticosteroids, difluprednate has been shown to be most effective for treatment of uveitic macular edema (33, 34). Periocular triamcinolone acetonide injections and intravitreal injections of triamcinolone acetonide or the dexamethasone implant are used in unilateral or bilateral uveitis as monotherapy, or in combination with systemic immunomodulatory therapy to achieve a more rapid resolution of the edema (35–38). The most common complications of local corticosteroid treatments are elevated intraocular pressure and cataract. Systemic use of corticosteroids is indicated mainly for bilateral macular edema and should be used for a limited time due to the extensive list of side effects (39). A variety of conventional and biologic systemic immunomodulatory agents are used in the treatment of non-infectious uveitis, and a number may have activity against macular edema even in the absence of apparent intraocular inflammation (40). Intravitreal injections of VEGF agents, such as bevacizumab and ranibizumab, or methotrexate may be used in refractory uveitic macular edema or when corticosteroid therapeutics are contraindicated (41–43).

Other approaches have been used to treat macular edema. Non-steroidal anti-inflammatory drugs have an adjunct role in the treatment approach, and mostly are used for pseudophakic macular edema (44). Acetazolamide, an inhibitor of carbonic anhydrase (CA), is occasionally employed in cases of failed corticosteroid therapy, but sometimes is used as primary therapy for pseudophakic macular edema or macular edema associated with retinal dystrophies (44). Pars plana vitrectomy is mostly reserved for treating mechanical factors contributing to macular edema, such as epiretinal membranes and vitreoretinal traction. It is the last resort for the treatment of macular edema without mechanical factors, owing to its inherent risks, which include retinal breaks and detachment, vitreous hemorrhage, cataract, glaucoma and hypotony, and limited data on outcomes (44–47).

## 3. Mechanisms of macular edema

### 3.1. Fluid forces driving macular edema

Macular edema is the outcome of the failure of mechanisms maintaining fluid homeostasis in the macula. The movement of fluid across capillaries throughout the body is classically described by Starling forces. A hydrostatic pressure gradient between the intravascular space and the tissue extracellular space tends to drive fluid into the tissue, and this is opposed by a colloid osmotic (oncotic) pressure gradient which tends to draw fluid out of the extracellular space into the blood vessels. A recent review by Cunha-Vaz discusses macular edema formation in the context of classic Starling forces (48). A relative increase in hydrostatic pressure in vessels versus the retinal tissue and a relative increase in oncotic pressure in the retinal tissue versus the vessels are the two forces that drive edema. Of these, macular edema in most diseases is thought to result from increased tissue oncotic pressure due to aberrant accumulation of solutes in the retinal extracellular space. The compliant nature of the retinal tissue, which can expand unimpeded into the vitreous, renders it particularly susceptible to edema as rises in tissue oncotic pressure readily incur increases in tissue volume. It should be noted that updated models of capillary filtration predict the tissue oncotic pressure to have limited effect on the rate of fluid flow across capillaries (49, 50). The relevance of these models to the retina is debatable, however, as in the absence of a known lymphatic system, the net movement of water between the retinal tissue and the blood must be zero or slightly outward due to metabolic water production, which counters the conjecture in these models that fluid flow is inward leading to a protected subglycocalyx space.

Tissues other than the macula are also susceptible to edema in unrelated conditions including pulmonary edema, cerebral edema and lymphedema. In all cases, the fundamental principle of imbalanced hydrostatic and osmotic forces applies. Pulmonary edema and lymphedema are quite distinct entities from macular edema, involving different barriers and unique underlying mechanisms. Cerebral edema, however, shares a degree of similarity with macular edema at the molecular level. It can be caused by disruption of the blood–brain barrier (BBB), the brain’s counterpart to the BRB, which is similar in its structural composition. Key molecules that disrupt both BRB and BBB integrity include VEGF (51) and matrix metalloproteinases (52), among others. The involvement of these factors in BRB disruption is discussed below. Shared mechanisms indicate the possibility of overlapping treatment opportunities.

It is unclear why the macula is uniquely vulnerable to edema as opposed to other regions of the retina. In their 2018 review, Daruich et al. described several hypotheses, including that disruption of junctional interactions between highly elongated Müller cells and photoreceptor axons in the perifoveal Henle’s layer may permit accumulation of protein, which is normally excluded from this region (2). They also proposed that a glymphatic system involving Müller glia may play a major role in retinal fluid and solute homeostasis, and this is discussed below. While the existence of such a system remains to be demonstrated, the unique cellular architecture of the fovea and perifovea likely challenges the mechanisms of solute clearance and fluid homeostasis that suffice to counter the abnormal accumulation of fluid in other regions of the retina. Mechanistic studies of the macula are difficult given that only humans and some primates possess a macula. Many studies of mechanisms underlying macular edema are performed in rodents or using cultured cells *in vitro*, and it is not apparent *a priori* which mechanisms will translate to the situation in the human macula.

The dysregulated entry of solutes to the retina occurs due to disruption of the BRB, and this is the main focus of most efforts to understand the mechanisms of this condition. An array of molecules and other insults have been described to modulate function of the BRB, and those selected for discussion in this review mainly relate to current treatments.

### 3.2. Cellular composition and structure of the blood-retinal barrier

The BRB is a group of structures in the eye that separates the blood from the neural retina and retinal extracellular space. There are two components: the inner BRB, and the outer BRB. The inner BRB is based around the retinal endothelial cells (REC) which line the retinal blood vessels, and the outer BRB is formed at the level of the retinal pigment epithelial (RPE) cells. As there can be non-selective bidirectional diffusion of solutes between the retinal extracellular space and the vitreous, barrier processes between the vitreous and blood vessels at the ciliary body might also be considered to form part of the BRB, although this region is not grouped in either the inner or outer BRB.

#### 3.2.1. Structure of the inner blood retinal-barrier

**Figure 2:**
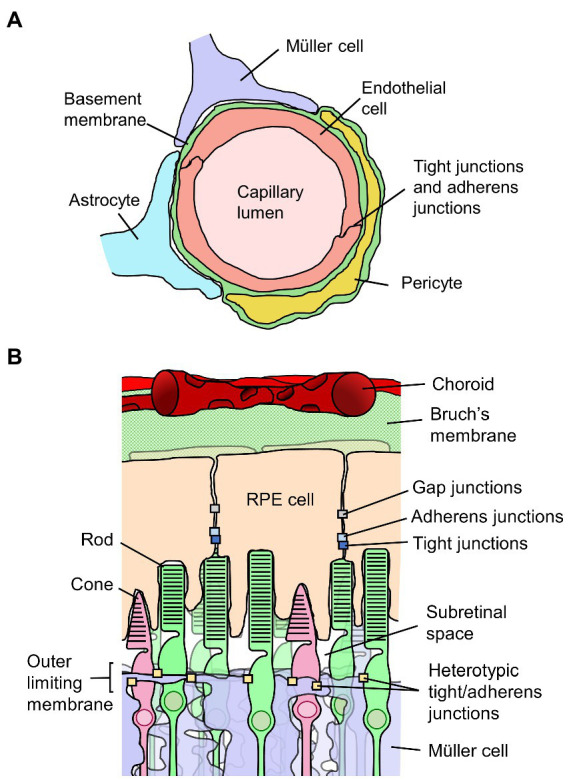
Diagrams of the inner and outer blood-retinal barriers (BRB). **(A)** Schematic cross-section of a retinal capillary indicating the location of junctions between retinal endothelial cells which form the inner BRB, along with surrounding cell types. **(B)** Diagram of the outer portion of the retina and choroid, indicating the junctions between retinal pigment epithelial (RPE) cells which form the outer BRB, along with surrounding structures.

The walls of the retinal vessels form the inner BRB. The luminal surface of retinal blood vessels is uninterruptedly lined by a squamous layer of RECs, and the membranes of adjacent RECs are held together by a continuous network of tight junctions. Figure 2A depicts the inner BRB structure. The RECs are not fenestrated, and they contain few pinocytic vesicles. The RECs and their junctions form a barrier that facilitates the regulated passage of solutes between the blood and the retina (53). Basement membrane completely covers the abluminal surface of the RECs. Evidence collected *in vivo* indicates the basement membrane does not directly constitute a barrier (53–55), although some evidence indicates it can impede diffusion of molecular tracers *in vitro* (56). Larger vessels have additional layers of smooth muscle, basement membrane, and adventitia, differing in structure depending on the size and type of vessel (57).

Several cell types are situated in close proximity to the retinal vessels. Pericytes are embedded within the basement membrane of the abluminal wall of the vessels (57–59). In retinal capillaries, there is approximately a 1:1 ratio of endothelial cells to pericytes, meaning pericytes are abundant compared to most other capillary systems (58). Pericyte coverage of vessels is nonetheless discontinuous, so they do not form a barrier themselves. Müller glia, astrocytes and microglia directly border retinal vessels, separated by basement membrane from the RECs, pericytes or collagenous adventitia (60). Neurons are surrounded by Müller cells throughout the retina such that there is no direct contact between retinal vessels and neurons. Pericytes, astrocytes microglia and Müller cells are thought to contribute to the development of retinal vessels and the BRB (61–67). Whether these cell types are required to maintain the mature BRB is less clear and warrants further research. For example, depletion of pericytes from adult mice by induction of a genetically encoded diphtheria toxin gene did not induce BRB leakage (63), which countered the standing notion of the necessity of pericytes for BRB integrity (68). All of these cell types are, however, likely to be involved in a pathological or protective capacity in diseases affecting the BRB.

The supporting cell populations can influence fluid homeostasis *via* means other than direct effects on BRB integrity. As an example, Müller cells and astrocytes express the water channel aquaporin-4 (AQP4) in a distribution enriched at the endfeet bordering the vitreous and retinal vessels. Given its similar distribution to the Kir4.1 potassium channel, which plays a fundamental role in siphoning potassium from synapses to the blood and vitreous, AQP4 has been proposed as a mediator of ion-coupled water transport (69); however, this is uncertain with functional analysis indicating no interplay between Kir4.1 and AQP4 (70). Nevertheless, AQP4 is likely to be involved in retinal water homeostasis. In the brain, a so-called glymphatic system operates, whereby cerebrospinal fluid is circulated in the perivascular space between vessels and astrocyte endfeet rich in AQP4 (71). In the human macula, Daruich et al. observed channel-like structures outlined in the staining pattern of AQP4, particularly within Henle’s layer in the fovea, which is rich in AQP4 but absent of retinal vessels (2). They hypothesised that an AQP4-dependent fluid flow may exist to clear interstitial fluid from the central retina, with evidence of decreased AQP4 expression in the diabetic fovea. Direct evidence for this hypothesis is yet to be presented.

#### 3.2.2. Structure of the outer blood-retinal barrier

The outer BRB is not situated at a vascular wall, but rather is formed by the retinal pigment epithelium, a monolayer of RPE cells situated behind the photoreceptors and immediately opposed to Bruch’s membrane which separates it from the vascular bed of the choriocapillaris (54, 72). Figure 2B depicts the outer BRB structure. In contrast to the inner BRB, the vessels of the choroid have thin and highly fenestrated endothelial cells, and do not form a selective barrier (53, 54, 73). As such, the epithelium is responsible for maintenance of the regulated environment required for retinal function. As applies to other epithelia, RPE cells are polarized and connected to one another laterally by tight junctions located at the apical side of their adjoining surfaces (72). Adherens junctions are present as well, located just basally of and partially overlapping the tight junctions (74). The RPE cells are also connected and electrically coupled *via* gap junctions (75). The polarized distributions of various membrane transporters in RPE cells facilitate the healthy functioning of the retina and are involved in retinal fluid homeostasis. Water injected into the subretinal space is predominantly cleared across the epithelium by mechanisms dependent on ion transport (76–78).

The outer limiting membrane must also be considered when discussing the outer BRB. It is not a true basement membrane but is formed by tight-like and adherens junctions among Müller cells and the cell bodies of photoreceptors (2). The pore size of the outer limiting membrane is small, restricting the passage of large molecules including albumin (79), so the membrane may be an important factor in macular edema development or in the type or location of edema. For instance, it may play a key role in the development or localization of subretinal edema.

Although the choroid sits outside the site of the outer BRB, together with Bruch’s membrane, it may still influence fluid homeostasis across the barrier. For instance, the hydraulic conductivity of Bruch’s membrane decreases with age (80), which is a possible factor in the accumulation of subretinal fluid in exudative age-related macular degeneration (81). Changes to the choroid are also likely to affect processes of retinal fluid absorption across the RPE cells. There are choroidal changes in diabetes, including choroidal vascular loss, choroidal neovascularization, and altered subfoveal choroidal thickness, which are reported to correlate with edema (82, 83). There is conflicting data on the subfoveal thickness changes, which is discussed in a report by Lutty (84). A precise mechanistic link between these changes and formation of retinal edema is yet unclear. Choriocapillaris loss may decrease the capacity for local fluid clearance or induce local hypoxia and promote expression of VEGF (85), with subsequent neovascularization and elevated vascular permeability. Serum leakage resulting from neovascularization may influence RPE cell transport processes and osmotic balance.

#### 3.2.3. Molecular composition of the intercellular junctions of the blood-retinal barrier

The key structures responsible for paracellular BRB integrity are the junctional interactions between the RECs or RPE cells. Junctional complexes connect adjacent cells completely and greatly limit the passage of solutes through the gaps between cells. Generally considered most crucial for this function are the tight junctions (zonulae occludens). Tight junctions are formed by the adherent interactions of specific protein complexes on adjacent cells. These complexes comprise various transmembrane and intracellular proteins, and their composition varies depending on the cell type or context. In general, the extracellular regions of transmembrane proteins adhere in a homo- or heterotypic manner to tight junction proteins on adjacent cells. Common transmembrane proteins include occludin and other MARVEL domain proteins, claudins, and JAMs. Occludin is present at tight junctions in both RECs and in RPE cells. Claudin −1, −2 and − 5 are the predominant claudins at REC tight junctions (86). Claudin-19 is the predominant claudin at RPE tight junctions, with claudin-3 and -10 also expressed (87, 88). The cytoplasmic regions of the transmembrane proteins contact intracellular proteins which link the tight junctions to the actin cytoskeleton, and which participate in cell signaling. Common intracellular proteins include the zonula occludens (ZO) proteins, ZO-1, −2 and − 3. Tight junctions are dynamic, with certain proteins diffusing within the junction or undergoing exchange between cytosolic and membrane-localised pools (89). In addition to restricting paracellular passage of solutes, tight junctions contribute to processes of cell polarity, especially in epithelial cells, where they are located at the boundary between apical and basolateral plasma membrane domains (90), although mature tight junctions are not necessary for polarization (91).

Adherens junctions (zonulae adherens) are another structural component that joins adjacent cells in the inner and outer BRBs. These junctions are also formed by interactions of like proteins between two cells. The nectin family and cadherin family are major adhesion proteins at adherens junctions. Vascular endothelial (VE)-cadherin is the main player in endothelial cells (92). In RPE cells, it has been reported that placental (P)-cadherin is most highly expressed – at least at the mRNA level – which differs from most epithelia, as epithelial (E)-cadherin usually predominates (93). The cytoplasmic regions of cadherins interact with members of the catenin family, p120-, α- and β-catenin which link to and locally modulate the actin cytoskeleton, and participate in cell signaling and transcriptional control (94). The nectins link to the actin cytoskeleton *via* afadin. While adherens junctions are not classically regarded as key mediators of the barrier function in barrier epithelia and endothelia, there is functionally important interplay between adherens junctions and tight junctions (95). In fact, intravitreal injection of an anti-VE-cadherin antibody in rat eyes reportedly increased vascular permeability by nearly five-fold, demonstrating a direct importance of adherens junctions in BRB function (96).

The composition of tight junctions and adherens junctions and roles of the proteins involved in these cell–cell contacts have been reviewed by others (see references (72, 94, 97, 98)). Here, mechanisms of disruption of the junctions, as well as disruption of the transcellular route of BRB dysfunction are discussed as related to macular edema.

### 3.3. Blood-retinal barrier dysfunction: routes of solute transit and molecular mediators

Dysfunction of the BRB underlies development of macular edema. Loss of selectivity of the exchange of molecules between the blood and retinal tissue at the level of either the inner or outer BRB permits entry of proteins and other solutes to the retinal tissue. Leakage is usually evident well prior to macular edema formation. It is likely that edema eventuates when the severity of leakage overcomes solute and fluid clearance mechanisms (99). The rate of clearance of solutes from the subretinal space is inversely proportional to their size (100). Injection of variously sized, tagged dextran tracers into patients with uveitic macular edema showed rates of leakage roughly proportional to the size of the tracer. Sizes up to 20 kDa leaked into the macula, but 150 kDa did not (101). These data indicate that, while infiltration of larger solutes likely poses a greater challenge for clearance, complete BRB breakdown permitting non-selective entry of the largest solutes is not necessary for edema to form.

Solutes can enter the retina across the dysfunctional BRB in two ways: paracellularly or transcellularly. The paracellular route involves passage of solutes *via* the gaps between cells due to disruption of the junctional contacts. The transcellular route involves increased vesicular transport of solutes across cells and/or increased passive diffusion due to elevated membrane permeability. The routes of breakdown may differ between diseases, and both routes may be involved (102).

The widespread nature of BRB leakage in many diseases suggests that soluble factors likely play a role. Many growth factors and inflammatory cytokines have been found elevated in the intraocular fluids of patients with diseases complicated by macular edema, and there is evidence that several act to disrupt BRB integrity. These molecules can drive BRB breakdown in multiple ways. They may act directly on the RECs or RPE cells to increase permeability by affecting the expression or regulation of junctional molecules (103, 104); they may act on other retinal cell types to further induce their own expression or that of other inflammatory mediators, facilitating a persistent inflammatory state; or they may attract and promote adhesion of leukocytes to the retinal vasculature (105, 106) which then induce BRB breakdown directly or further release inflammatory mediators (107, 108). The relative importance of various inflammatory and vasoactive molecules, their cellular sources, and the precise sequence of events leading to BRB breakdown and macular edema is not yet clear, and likely differs between diseases and individual patients. A selection of mediators of BRB dysfunction of relevance to current macular edema treatments is discussed below.

#### 3.3.1. Vascular endothelial growth factor-A and placental growth factor

Vascular endothelial growth factor-A is a major mediator of pathological vascular changes in several ocular diseases. The presence of VEGF is higher in the ocular fluids of patients with diabetic retinopathy and central retinal vein occlusion than in healthy controls (109). It is upregulated in experimental autoimmune uveoretinitis (110). Macular edema in retinal vascular occlusion is driven by VEGF in many but not necessarily all cases (111). Intravitreal levels of VEGF and IL-6 correlate with the severity of macular edema and the size of the non-perfusion area (112, 113). This is consistent with the observations that VEGF expression is induced by hypoxia *in vitro* in various retinal cell types, and hypoxia-conditioned medium stimulates proliferation of RECs (114).

Vascular endothelial growth factor-A increases permeability of bovine REC monolayers *in vitro* (115). Mechanistically, VEGF activates protein kinase C (PKC)β which directly phosphorylates occludin, targeting it for ubiquitination (116, 117). This alters trafficking of occludin and its normal localization at tight junctions (116). Inhibition of PKC, transfection of a dominant negative PKC, or mutation of the phosphorylated residue of occludin counters VEGF-induced permeation of bovine REC monolayers (115, 118). Occludin content is lower in retinas of diabetic rats, and phosphorylation of occludin is elevated (118, 119). Intravitreal injection of VEGF induces fluorescein leakage from retinal vessels in rats *in vivo*, and this is almost completely abolished by inhibition of PKCβ (120). However, phase 3 studies of a PKCβ inhibitor showed limited benefit in treatment of diabetic retinopathy (121). There is a wealth of evidence supporting the mechanism of VEGF-induced alteration of occludin distribution leading to functional changes in tight junctions *in vitro*. When assessed at the ultrastructural level, however, REC tight junctions often appear non-leaky in models of diabetes, indicating other mechanisms of BRB breakdown are also likely at play (122–124). Intravitreal injection of VEGF in cynomolgus monkeys increased vascular permeability without opening tight junctions (125). Instead, there was a stark change in the distribution of pinocytic vesicles in RECs, said to indicate an increase in vesicular transport of macromolecules from the vascular lumen to the retinal extracellular space. It is possible that non-responsiveness to VEGF blockers may also occur where outer BRB breakdown is apparent. Both VEGF and anti-VEGF treatments are reported to have minimal effects on RPE cell tight junctions *in vitro*, meaning other mechanisms may need to be targeted (87). Outer BRB breakdown may also limit the proposed ability of the epithelium to remove VEGF from the subretinal space (87).

In endothelial cells, adherens junction components including VE-cadherin, p120- and β-catenin, and plakoglobin undergo tyrosine phosphorylation in response to VEGF which coincides with increased permeability (126). A more recent study showed that VEGF induces activation of the small GTPase, Rac, then p21-activated kinase (PAK), which phosphorylates a specific serine residue on VE-cadherin leading to its internalization, disassembly of the adherens junction, and increased permeability (127).

Placental growth factor is an angiogenic growth factor in the VEGF family, which may also be involved in promoting BRB permeability. It is greatly upregulated in retinae of Akita diabetic mice, and its knockout in this model protects against retinal capillary leakage and degeneration (128). The PlGF gene deletion not only abrogated the decrease in expression of ZO-1 and VE-cadherin in diabetic mice, but elevated their expression relative to non-diabetic controls. Expression of these junctional molecules correlated with expression of two putative regulators, sonic hedgehog and angiopoetin-1, the latter of which has been shown to protect against BRB breakdown (129). Hypoxia-inducible factor (HIF)-1α and VEGF expression were upregulated in Akita mice, and this was countered by PlGF knockout.

Clearly, VEGF plays a major role in driving progression of retinal diseases and macular edema, which is irrefutably evidenced by the broad utility of anti-VEGF therapies. Targeting VEGF with intravitreal injections of monoclonal antibodies or high-affinity decoy receptors are effective means of treating most diseases complicated by macular edema, including wet age-related macular degeneration (130), diabetic macula edema (131, 132), and macular edema caused by CRVO (133), BRVO (134) and uveitis (135). However, there are concerns of ocular and systemic toxicity in targeting VEGF, and long-term frequent reinjection is required (136–138). Also, the fact that there are cases resistant to anti-VEGF treatment suggests other pathways may also be involved in these diseases.

#### 3.3.2. Tumor necrosis factor-α and interleukin-1β

Tumor necrosis factor-α is a master inflammatory cytokine which has been shown to increase barrier permeability of REC monolayers *in vitro* (139). When injected into the vitreous in rabbits, TNF-α increased the proportion of REC tight junctions that appeared open, and albumin was detected within these junctions, indicating leakiness (140). Larger vesicles also stained positively for albumin in RECs in treated eyes, but not controls. These results indicate breakdown of both transcellular and paracellular pathways. Interestingly, 24 h after injection, TNF-α-induced tight junction changes had reversed, a promising sign for a potential treatment target. When injected into the vitreous in rats, it also induced barrier leakage, and the effect of injection recovered after 7 days (141). Huang et al. used two mouse models of diabetes to assess the pathological role of TNF-α (142). Vascular leakage late in disease was completely prevented by TNF-α knockout. Interestingly, these observations did not correlate with the expression of TNF-α in the control diabetic mice, as TNF-α expression was not elevated in mid or late diabetic retinae. Instead, it was proposed that early expression of TNF-α might initiate a chain of events leading to increased apoptosis of retinal cells and BRB breakdown in later stages. In bovine REC monolayers, TNF-α treatment decreased expression of ZO-1 and claudin-5 but increased expression of occludin (139). It disrupted the normal border staining pattern of all three. The increase in barrier permeability induced by TNF-α was prevented by dexamethasone or by an inhibitor of PKCζ. The PKCζ inhibitor also prevented BRB disruption induced by intravitreal injection of TNF-α in rats *in vivo*.

Treatment of human fetal RPE cells *in vitro* with TNF-α decreased resistance of monolayers by 80% (143). Interestingly, in a particular serum-free medium, this effect was only observed when TNF-α was applied to the apical side of the monolayer. A clear molecular mechanism for TNF-α-mediated barrier disruption was elusive, with no changes observed in levels of several junctional molecules tested, but it was suggested that TNF-α may induce changes in tight junction tortuosity.

Anti-TNF-α monoclonal antibodies are sometimes used in the treatment of non-infectious uveitis (144). They show high efficacy, although corticosteroids and conventional immunosuppressive drugs are generally the first-line treatment (145). Tumor necrosis factor-α has essential roles as an infection-fighting agent, so patients require close monitoring due to risk of developing infections, and increased risk of cancer may also be a concern. Some data suggest TNF inhibitors used for treatment of non-ocular diseases could even worsen or increase the risk of developing uveitis, a point which will require larger-scale studies to resolve (146).

Along with TNF-α, another master inflammatory cytokine which is of increasing interest is IL-1β. Similar to TNF-α, when injected into the rabbit vitreous, IL-1β increases the number of open, leaky tight junctions (140). It also increases the permeability of REC monolayers *in vitro* (139). In streptozotocin-diabetic rats, retinal IL-1β expression increased between 1.5 and 4.5 months of diabetes (147). IL-1β mRNA is induced by high glucose in bovine RECs *in vitro* (147). It induces its own expression in RECs, Müller cells and brain astrocytes *in vitro*, which is prevented by inhibition of protein kinase C (147). The effects of IL-1β may be due to elevated oxidative stress, decreased expression of occludin, ZO-1 and claudin-5, and altered distribution of VE-cadherin (148). Interleukin-1β may be a promising target for new therapeutics: a receptor antagonist (anakinra), a decoy receptor (rilonacept), and a neutralizing antibody (canakinumab) are already in clinical use for other indications.

#### 3.3.3. Interleukin-6

Interleukin-6 is a cytokine intimately linked with inflammatory and autoimmune disease, and is a promising target for treatment of non-infectious uveitis (149). Circumstantial evidence implicates IL-6 in diseases leading to macular edema. It is elevated in patients with uveitis (150). It is elevated in diabetic macular edema (151) and correlates with macular thickness (152). IL-6 is also upregulated in patients with central retinal vein occlusion, especially those with ischemia (112). Its expression often correlates with that of VEGF.

Experimental evidence for the role of IL-6 in disease is less clear at present. IL-6 did not induce noticeable barrier permeability to mannitol when injected into the vitreous in rats (141). *In vitro*, IL-6 treatment disrupted the barrier formed by the ARPE-19 RPE cell line, as evidenced by increased permeability to 40 kDa dextran and decreased electrical resistance (153). Distribution of ZO-1 at the borders was disrupted, suggesting tight junction dysfunction may be involved. In contrast, IL-6 had no effect on the permeability of human REC monolayers (153). It can, however, reportedly induce permeability of non-REC monolayers *in vitro* (104), although in some cases the effect requires exogenous addition of IL-6 receptor (154). Together, these data suggest IL-6 may be especially active on RPE cells in inducing permeability.

As opposed to or in addition to acting directly on the barrier cells, IL-6 may act *via* less direct means, promoting inflammation to indirectly facilitate disease progression. Knockout of IL-6 or treatment with anti-IL-6 or anti-IL-6 receptor antibody ameliorates mouse experimental autoimmune uveoretinitis, a model of human non-infectious uveitis (155–157). A major mechanism underlying the effectiveness of IL-6 blockade in this model is the prevention of differentiation and expansion of T helper 17 cells (155, 156). Clinical trials of biologic drugs that target IL-6 signaling – such as tocilizumab and sarilumab – for macular edema are ongoing, with initial positive results (149, 158).

#### 3.3.4. Matrix metalloproteinases

Growth factors and inflammatory cytokines are not the only targets of treatments for macular edema. Corticosteroids provide effective treatment for macular edema resulting from various diseases, including diabetes mellitus, central and branch retinal vein occlusions, and uveitis (159–161). These drugs are very broad-acting and have potent anti-inflammatory effects. One of many mechanisms by which they may act is the suppression of matrix metalloproteinase (MMP) expression and activity (162). The MMPs are a family of endopeptidases which degrade extracellular matrix and basement membranes among other macromolecules, and they have been suggested to play a role in progression of diabetic retinopathy (163).

Matrix metalloproteinases, MMP2, MMP9 and MMP14, are upregulated in the retinal vessels at the RNA level in streptozotocin-diabetic rats (96, 164). Retinal vascular permeability is induced in this model, and this could be prevented by intraperitoneal injection of a broad spectrum MMP inhibitor. Mechanistically, MMP inhibition prevented the loss of surface staining of VE-cadherin on RECs. The elevated presence of advanced glycation end products was proposed to underly the heightened activity of MMPs in the diabetic model. MMPs may play a role in modulating both inner and outer BRB integrity, with treatment of the ARPE19 RPE cell line, as well as bovine RECs, with recombinant MMP2 or MMP9 decreasing electrical resistance of the monolayers (164). In this study, the mechanism was proposed to be degradation of occludin and tight junction alterations (164).

#### 3.3.5. Carbonic anhydrase

Carbonic anhydrase inhibitors, including acetazolamide, have a history of use for treating macular edema, especially that caused by retinitis pigmentosa and diabetic retinopathy (165–167). Isoforms CA-I and CA-II were found to be elevated in the vitreous of patients with proliferative diabetic retinopathy (168). Injection of CA into rat vitreous induced vessel leakage which was prevented by co-injection of CA inhibitors. It is possible that the elevated intravitreal CA derives from intraocular hemorrhage, as injection of lysed red blood cells recapitulated vessel leakage, and this can be prevented by CA inhibition. The effect of CA on BRB permeability was found to be mediated by the kallikrein-kinin system. Notably, injection of VEGF resulted in a similar magnitude increase in vascular permeability as injection of CA, VEGF did not act *via* the same pathway, and the effect of CA and VEGF together was additive, suggesting dual treatment could prove useful. In addition to the above mechanism, CA inhibitors also act on membrane-bound CA on RPE cells and increase resorption of subretinal fluid across the epithelium (169–171). The precise mechanism by which this works is an area of continuing research. A recent mathematical model of ion transport across the RPE cell barrier predicted this behavior (78).

### 3.4. Dysfunction of the transcellular arm of the blood-retinal barrier

As is evident from this discussion, molecular studies of BRB breakdown frequently focus on changes in expression and localization of junctional proteins, which are key players in paracellular BRB integrity. There is ample evidence that many molecules elevated in retinal diseases affect paracellular integrity. However, the ultrastructural studies that have been performed have indicated transcellular routes of BRB dysfunction are likely equally important as paracellular dysfunction. There are several means of transcellular passage (92), including simple diffusion – which is minimal for hydrophilic molecules – ion channels for salts and small molecules, specific transporters for amino acids and metabolites, and small vesicles termed caveolae, which when dysregulated can facilitate non-selective entry of proteins and solutes. Small vesicles are scarce in RECs compared to non-barrier endothelia, and this is reportedly a key feature that contributes to the inner BRB (172). Electron microscopy shows that vesicles are predominantly located at the abluminal side of the RECs, which has been suggested to indicate most transport occurs in the retina-to-blood direction (125). Caveolae are the best studied means of trans-endothelial vesicular transport across the BRB, reviewed by Klassen et al. (92). Caveolin-1 is the predominant protein constituent of caveolae. Both its knockout (173), and its overexpression (174) have been reported to disrupt BRB function. Caveolin-1 and caveolae are likely multifunctional and are only beginning to be understood in the context of the BRB.

Based on the ultrastructural localization of albumin in animal models of diabetes and in diabetic human retinas, tight junctions between RECs or RPE cells rarely appear disrupted, with albumin leakage instead suggested to be predominantly transcellular *via* vesicles or *via* diffusion through hyperpermeable plasma membranes due to degenerative changes in RECs or RPE cells (122–124, 175). In a rat model of experimental autoimmune uveoretinitis, leakage of albumin into the retinal tissue and presence of albumin in REC vesicles and RPE vacuoles was evident prior to ultrastructural evidence of disruption of tight junctions (140). Tight junctions did subsequently open and also permit passage of albumin. Tight junctions also appear to remain intact in the face of acute ischemic or chemical injury despite significant damage to RECs and transcellular leakage of tracers, although leakage in this setting is likely due to gross damage to the RECs rather than dysregulated vesicle transport (55). The potential for the transcellular route of BRB transit to account for vessel leakiness is also apparent in the process of inner BRB development. Newly sprouting vessels in later stages of development are leaky to tracers, but possess functional tight junctions (176). Leakage occurs through RECs *via* vesicles, and the fully functional BRB only forms upon suppression of transcytosis (176).

It is not yet clear which route of breakdown is responsible for the greatest portion of solute entry during BRB dysfunction, and the situation in the human macula may differ to animal models and to peripheral regions of the retina. Paracellular and transcellular routes may both be involved in many cases of macular edema.

## 4. Conclusion

Current treatments for macular edema show effectiveness in some, but not all situations. The continued use of broad acting treatments such as corticosteroids as first-line agents in spite of significant side effects highlights the incomplete understanding of the mechanisms underlying disease and the need for continuing research. Ongoing efforts to understand mechanisms governing regulation of junctional complex molecules in response to growth factors and inflammatory cytokines may clarify the contexts in which these mediators are involved. Major holes in current knowledge also include the reason why the macula is uniquely vulnerable to edema, as well as the mechanisms underlying dysfunction of transcellular integrity. Dysfunction of the BRB has a diverse spectrum of causes, in addition to those presented here. Though macular edema may be a common outcome of BRB dysfunction, the disparate causes of BRB dysfunction may require a battery of options such that treatment may be tailored to the cause of each case.

## Author contributions

KW and JS conceptualized the review and reviewed and edited the manuscript draft. CH extensively reviewed the literature and drafted Sections 1. Introduction, 3. Mechanisms of macular edema, and 4. Conclusion. LF extensively reviewed the literature and drafted Section 2. Clinical overview of macular edema and Figure 1. All authors read and approved the final manuscript.

## Funding

This work was supported by a Research Grant from Macular Disease Foundation Australia.

## Conflict of interest

The authors declare that the research was conducted in the absence of any commercial or financial relationships that could be construed as a potential conflict of interest.

## Publisher’s note

All claims expressed in this article are solely those of the authors and do not necessarily represent those of their affiliated organizations, or those of the publisher, the editors and the reviewers. Any product that may be evaluated in this article, or claim that may be made by its manufacturer, is not guaranteed or endorsed by the publisher.
